# Engineering In vitro Models: Bioprinting of Organoids with Artificial Intelligence

**DOI:** 10.34133/cbsystems.0018

**Published:** 2023-03-29

**Authors:** Hyungseok Lee

**Affiliations:** ^1^ Department of Mechanical and Biomedical Engineering, Kangwon National University (KNU), 1, Kangwondaehak-gil, Chuncheon-si, Gangwon-do, Republic of Korea.; ^2^ Department of Smart Health Science and Technology, Kangwon National University (KNU), 1, Kangwondaehak-gil, Chuncheon-si, Gangwon-do, Republic of Korea.

## Abstract

In the last decade, organoids have gained popularity for developing mini-organs to support advancements in the study of organogenesis, disease modeling, and drug screening and, subsequently, in the development of new therapies. To date, such cultures have been used to replicate the composition and functionality of organs such as the kidney, liver, brain, and pancreas. However, depending on the experimenter, the culture environment and cell conditions may slightly vary, resulting in different organoids; this factor significantly affects their application in new drug development, especially during quantification. Standardization in this context can be achieved using bioprinting technology—an advanced technology that can print various cells and biomaterials at desired locations. This technology offers numerous advantages, including the manufacturing of complex three-dimensional biological structures. Therefore, in addition to the standardization of organoids, bioprinting technology in organoid engineering can facilitate automation in the fabrication process as well as a closer mimicry of native organs. Further, artificial intelligence (AI) has currently emerged as an effective tool to monitor and control the quality of final developed objects. Thus, organoids, bioprinting technology, and AI can be combined to obtain high-quality in vitro models for multiple applications.

Organoid technology has recently received considerable attention owing to its ability to create mini-organs and therefore support studies on organogenesis, disease modeling, and drug development [1]. To date, this technology has been employed to create lab-grown miniature versions of organs—such as kidney [2], liver [3], brain [4], and pancreas [5]—that exhibit close resemblance to real organs in terms of composition and functionality. The ability of organoids to self-organize and assemble makes these structures useful for modeling human organ development [6], a process that cannot be studied in animal models owing to interspecies differences. For drug approval by competent authorities, in vivo studies screening for novel drug compounds, testing efficacy, and toxicity are necessary [7]. Following proper validation as pre-screening systems for novel drugs, in vitro studies in organoids can substantially reduce the number of animals used during drug testing [8]. Furthermore, the ability of organoids to mimic human pathologies at the organ level can counteract the lack of appropriate animal disease models [9]. Patient-derived organoids can provide the means to develop personalized approaches and lead to advancements in precision medicine [10]. They can be employed to select appropriate drugs for treatment of patients with genetic diseases or cancer, to predict response to drugs, and to choose better therapeutic options for each individual or groups of individuals.

Despite the wide range of possible applications of organoids, they are under-researched. For example, during organoid production, in addition to the sophisticated simulation of internal body flow conditions, maintaining the reproducibility of different cell types, such as vascular cells, and predicting the extent of organoid formation are important [11]. Although organoid technology can be successfully applied for disease modeling, developing a standardized organoid model for drug development is difficult owing to current technical limitations: Depending on the experimenter, the culture conditions and cell conditions may vary, resulting in variations in the organoids produced, which is a factor that significantly affects their application in new drug development, especially during quantification [12]. Researchers have successfully cultured and maintained organoids embedded in matrix for extended periods of time. However, one of the biggest challenges in organoid culture is delivering nutrients and gas exchange, especially as the organoids grow [13]. Through perfusion, nutrients can be continuously replenished and waste can be removed; this process is therefore more increasingly being utilized in organ-on-chip cultures to maintain all the nutrients, growth factors, and metabolites in a constant equilibrium once optimized [14]. However, the reliance on passive diffusion to supply nutrients or oxygen within the organoid mass can lead to variations between the metabolic status of cells within a spheroid mass and that of the actual tissue of interest.

Biofabrication becomes more challenging for organoids of increasing complexity in terms of cellular and extracellular matrix compositions. Among various other techniques (such as microplates and microfluidics), inkjet, laser-assisted, and extrusion methods of bioprinting have emerged as powerful tools for engineering biological structures [15]; these methods offer the capabilities to develop organoids with distinct cell densities and mechanical properties. Although all bioprinting techniques offer various advantages, extrusion-based bioprinting is one of the most widely used techniques owing to its excellent abilities when using multiple cell types and biomaterials [16,17]. Therefore, extrusion-based bioprinting has been studied to create organoid structures that maintain their complex biological three-dimensional (3D) shapes [18]. Bioprinting can be used not only for printing biological components but also for standardization and quality control during organoid production to minimize human intervention. Although few studies have attempted to apply extrusion-based bioprinting technology for simple organoid production [19,20], further research is required. In addition to expanding the applications of bioprinted organoids, future studies can focus on improving the resolution of the bioprinting process, especially of extrusion-based bioprinting. Through such improvements in various ranges, organoid bioprinting can potentially be applied to generate vascularized organoids with perfusion networks or organoid-based large-volume tissue for tissue engineering, which can overcome the limitations in delivering nutrients and oxygen to organoids. Another major concern is bioink development—the shear stress–induced cell damage owing to high-viscosity bioink leads to the production of organoids with undesirable characteristics.

Multiple studies have been conducted in the field of data analysis for medical technology using artificial intelligence (AI) [21]; however, technologies that combine organoids, bioprinting, and AI are limited. The application of AI in bioprinting and organoids has gained recent research attention owing to its potential in the verification of products during fabrication. By monitoring cell cultures, more standardized cell sources in terms of viability, functionalities, etc., can be used for organoid development. During bioprinting, improvement of the resolution and productivity of bioprinted structure is an essential process [22]. AI can also monitor the printed structure to ensure optimum size based on manufacturer requirements and can provide feedback for elaborated organoid printing, as printing resolutions can be critical for organoid engineering. Consequently, AI allows the real-time diagnosis and homogenization of organoids during bioprinting (Figure). In addition, it allows sophisticated disease modeling. The application of AI in bioprinting may also allow the prediction of the test results of various new drug combinations.
